# Healthy lifestyle changes favourably affect common carotid intima-media thickness: the Healthy Lifestyle Community Programme (cohort 2)

**DOI:** 10.1017/jns.2022.46

**Published:** 2022-06-13

**Authors:** Christian Koeder, Sarah Husain, Ragna-Marie Kranz, Corinna Anand, Dima Alzughayyar, Nora Schoch, Andreas Hahn, Heike Englert

**Affiliations:** 1 Institute of Food Science and Human Nutrition, Leibniz University Hanover, Am Kleinen Felde 30, 30167 Hanover, Germany; 2 Department of Nutrition, University of Applied Sciences Münster, Corrensstr. 25, 48149 Münster, Germany

**Keywords:** Cardiovascular prevention, Plant-based diet, Lifestyle medicine, Carotid intima-media thickness, Cardiovascular disease, Cardiovascular health, Preventive medicine

## Abstract

Common carotid intima-media thickness (ccIMT) progression is a risk marker for cardiovascular disease (CVD), whereas healthy lifestyle habits are associated with lower ccIMT. The objective of the present study was to test whether a healthy lifestyle intervention can beneficially affect ccIMT progression. A community-based non-randomised, controlled lifestyle intervention was conducted, focusing on a predominantly plant-based diet (strongest emphasis), physical activity, stress management and social health. Assessments of ccIMT were made at baseline, 6 months and 1 year. Participants had an average age of 57 years and were recruited from the general population in rural northwest Germany (intervention: *n* 114; control: *n* 87). From baseline to 1 year, mean ccIMT significantly increased in both the intervention (0⋅026 [95 % CI 0⋅012, 0⋅039] mm) and control group (0⋅045 [95 % CI 0⋅033, 0⋅056] mm). The 1-year trajectory of mean ccIMT was lower in the intervention group (*P* = 0⋅022; adjusted for baseline). In a subgroup analysis with participants with high baseline mean ccIMT (≥0⋅800 mm), mean ccIMT non-significantly decreased in the intervention group (−0⋅016 [95 % CI −0⋅050, 0⋅017] mm; *n* 18) and significantly increased in the control group (0⋅065 [95 % CI 0⋅033, 0⋅096] mm; *n* 12). In the subgroup, the 1-year trajectory of mean ccIMT was significantly lower in the intervention group (between-group difference: −0⋅051 [95 % CI −0⋅075, −0⋅027] mm; *P* < 0⋅001; adjusted for baseline). The results indicate that healthy lifestyle changes may beneficially affect ccIMT within 1 year, particularly if baseline ccIMT is high.

## Introduction

Carotid intima-media thickness (cIMT) is a marker of pathological arterial wall changes (arterial injury)^(1–3)^. Often cIMT is referred to as subclinical atherosclerosis^(4)^, although this is controversially discussed^(2,3)^. Increased cIMT is a considerable public health concern^(5)^, and it has been estimated that the worldwide prevalence of increased cIMT (≥1⋅0 mm) is >25 % in individuals aged 30–79 years, equivalent to >1 billion individuals^(5)^.

Most frequently, cIMT is assessed in the common carotid artery^(6)^. Common cIMT (ccIMT) is an established risk marker for cardiovascular disease (CVD) such as myocardial infarction, sudden cardiac death^(7)^ and stroke^(8)^. Positive correlations have been shown between ccIMT and calcification scores of the coronary arteries and the aorta^(9)^ as well as between ccIMT and epicardial adipose tissue^(10)^.

The prospective assessment of ccIMT in clinical trials offers the possibility to test the effect of the intervention on the arterial structure, and this constitutes a more direct measure of artery health compared to serological markers, while at the same time avoiding invasive, complicated and very expensive procedures^(1,3,11)^. A recent meta-analysis of clinical trials, including dietary interventions, has shown that changes in ccIMT progression are able to predict changes in CVD risk, that even relatively small changes are relevant, and that ccIMT progression can thus be considered a suitable parameter for intervention studies^(12)^.

Current evidence indicates that a dietary pattern with a strong emphasis on healthy plant-based components, a high water intake and lower intakes of unhealthy plant-based foods and of meat is associated with lower ccIMT^(4,13–15)^.

Thus, the objective of the present study was to test if a healthy lifestyle intervention would beneficially affect ccIMT within 1 year (among other CVD risk markers^(16)^).

## Materials and methods

### Study design

The study was a non-randomised controlled trial, and ccIMT was assessed at baseline, 6 months and 1 year. The study had been planned with a duration of 2 years, but due to the COVID-19 pandemic we did not include the 1½-year time point (uneven time delays between groups; included in sensitivity analyses) or the 2-year time point (no assessment in the control group).

The intervention consisted of a lifestyle programme, and the control group received no intervention. Randomisation was not feasible, as described previously^(17)^. Both groups were recruited in two separate and comparable small towns in order to keep the participants of the control group unaware of the lifestyle recommendations given to the intervention group. The intervention group was recruited at a public market (February 2018) and by word of mouth, while the control group was recruited at a local public event (September 2018). The funding was provided for a specific time period, at relatively short notice and there were insufficient resources (time and staff) to recruit and start both study arms at the same time. Therefore, the intervention group started 6 months earlier (April 2018) than the control group study arm (October 2018), with equivalent follow-up intervals. The study was registered in the German Clinical Trials Register (DRKS; reference: DRKS00018775; www.drks.de).

### Participants

For the intervention and control groups, a total of 114 and 87 participants, respectively, were recruited. Participants were mostly middle-aged and elderly individuals from the general population in rural northwest Germany. As a community-based intervention, the only inclusion criteria were the physical and mental ability to participate and to be ≥18 years old. This study was conducted according to the guidelines laid down in the Declaration of Helsinki and all procedures involving human subjects were approved by the ethics committee of the Medical Association of Westphalia-Lippe and of the University of Münster (Münster, Germany; reference: 2018-171-f-S; approved 4 April 2018). Written informed consent was obtained from all subjects.

### Lifestyle programme

The lifestyle intervention has been described previously^(17)^. In brief, the intervention included an intensive phase (first 10 weeks; 14 seminars and 8 workshops) and a less intensive phase (monthly seminars). The focus of the intervention was on four areas of lifestyle change: a healthy, predominantly plant-based diet (strongest emphasis), physical activity, stress management and social health. Dietary recommendations were to consume more healthy plant foods (fruit, vegetables, whole grains, legumes, including soya foods, nuts, seeds and healthy oils) and to reduce the intake of unhealthy plant foods (added sugars, salt, refined grains, alcohol excess) and of animal-source foods (particularly meat)^(18)^. Participants also received a healthy lifestyle handbook, a recipe booklet and a laminated information sheet with an overview of the lifestyle recommendations^(17)^ (Supplementary material).

### Assessment of parameters

Measurements of ccIMT were conducted in accordance with the Mannheim consensus^(19)^, as described previously^(17)^ (Supplementary material). Dietary intake was assessed with semi-quantitative 3-d food protocols. Adherence to dietary recommendations was assessed with the plant-based diet index (PDI), healthful PDI (hPDI) and unhealthful PDI (uPDI)^(18)^ (as described previously^(16)^). As the association of the intakes of potatoes, fish, eggs and dairy with ccIMT are less certain, whereas hPDI includes these food groups as negatives, we also conducted a *post hoc* analysis with a modified hPDI (excluding the food groups potatoes, fish, eggs and dairy). Physical activity (in categories) and socio-demographic parameters were assessed using questionnaires.

### Study hypotheses

In terms of mean and max ccIMT, the study hypotheses were that the intervention would significantly decrease mean and max ccIMT (within-group and compared to control; from baseline to 1 year and from baseline to 1½ years). The two main hypotheses were regarding the between-group changes (mean and max ccIMT: 1-year changes). Any detected differences in the secondary end points mean and max ccIMT are considered exploratory.

### Sample size calculation

The primary outcome parameter of the study was body weight^(16)^, for which a sample size calculation was conducted, and a further sample size calculation was conducted for the secondary parameter ccIMT (as described previously^(17)^).

### Statistical analyses

Between-group differences in baseline characteristics were assessed using Fisher's exact test for categorical variables and independent *t* test and Mann–Whitney *U* test for normally and non-normally distributed continuous variables, respectively (as described previously^(17)^). To evaluate within-group changes, paired *t* test and Wilcoxon signed-rank test were used for normally and non-normally distributed data, respectively. Correlations were assessed with Spearman's rho correlations. All tests were two-sided.

To evaluate between-group differences in 1-year trajectories of ccIMT, a repeated measures analysis of covariance (ANCOVA) was used, with baseline ccIMT and potential confounders (especially age and sex) as covariates. Holm–Bonferroni correction was conducted to adjust for multiple comparisons. As it has been shown that baseline ccIMT is inversely associated with ccIMT change^(20)^ and ccIMT may thus more strongly decrease in those with high baseline ccIMT^(21)^, subgroup analyses were conducted, including only participants with high baseline mean ccIMT (≥0⋅800 mm). Analyses were based on unimputed data (complete case analysis, CCA). In sensitivity analyses, imputed data (last observation carried forward, LOCF) were used. Sensitivity analyses using log-transformed (lg10) ccIMT values were also conducted. All analyses were conducted using IBM SPSS Statistics (Version 25.0. Armonk, NY).

## Results

### Baseline characteristics

**Fig. 1.**
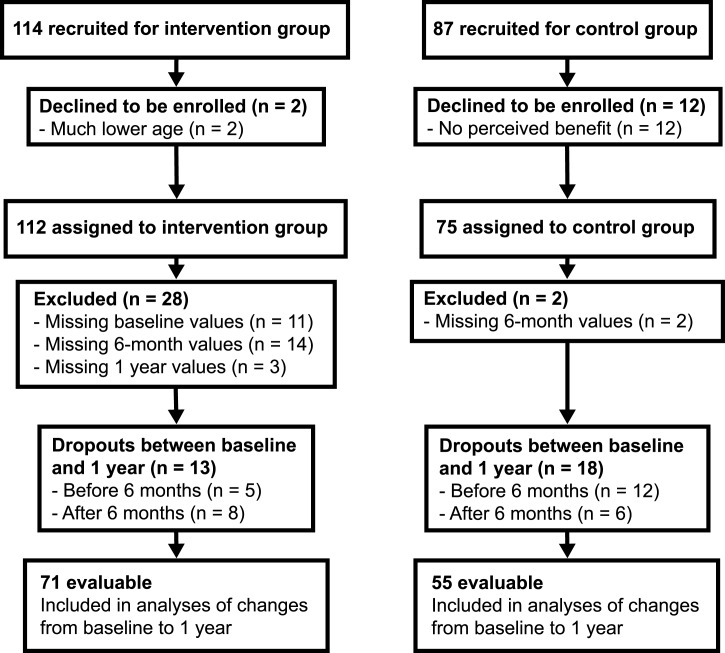
Flowchart of participants through the study.

For a total of 126 participants (intervention: 71; control: 55), ccIMT values were available for all three measurement time points (baseline, 6 months and 12 months). The flow of participants through the study is shown in Fig. 1.

At baseline, the intervention group had a significantly higher age and educational level (Table 1). Baseline hPDI was also significantly higher in the intervention group (by 4⋅3 [95 % CI 2⋅0, 6⋅5] portions/day; *P* < 0⋅001), while PDI and uPDI were not significantly different between groups. Frequency of intense physical activity was higher in the control group (*P* = 0⋅031) when assessed by categories, but physical activity (gentle, moderate and intense) was not significantly different between groups when assessed by minutes/week.

**Table 1.** Baseline characteristics of evaluable participants (CCA)

Characteristics	Intervention (*n* 71)		Control (*n* 55)		*P*-value*
	Mean or *n*	sem or %	Mean or *n*	sem or %	
Women, *n* (%)	50	70⋅4	34	61⋅8	0⋅344^a^
Age, years	59⋅4	1⋅0	55⋅3	1⋅3	**0⋅015**^b^
Mean ccIMT, mm	0⋅695	0⋅016	0⋅674	0⋅019	0⋅363^c^
Max ccIMT, mm	0⋅856	0⋅019	0⋅825	0⋅023	0⋅263^c^
Body weight, kg	81⋅4	2⋅2	84⋅4	2⋅5	0⋅269^c^
BMI, kg/m^2^	27⋅6	0⋅6	28⋅3	0⋅8	0⋅524^c^
WC, cm	98⋅2	1⋅8	97⋅5	2⋅1	0⋅957^c^
Overweight, *n* (%)	46	64⋅8	38	69⋅1	0⋅704^a^
Obese, *n* (%)	20	28⋅2	15	27⋅3	1⋅000^a^
Systolic BP, mmHg	132⋅0	1⋅7	133⋅2	2⋅1	0⋅658^b^
Diastolic BP, mmHg	79⋅9	0⋅9	80⋅1	1⋅3	0⋅998^c^
Pulse pressure, mmHg	52⋅1	1⋅4	53⋅1	1⋅6	0⋅626^b^
RHR, beats/min	68⋅1	1⋅3	69⋅4	1⋅2	0⋅508^b^
TC, mg/dl	207⋅5	4⋅7	208⋅9	6⋅0	0⋅863^b^
LDL-C (measured), mg/dl	133⋅0	4⋅4	140⋅6	5⋅9	0⋅292^b^
LDL-C (calculated), mg/dl	121⋅0	4⋅5	125⋅5	5⋅6	0⋅526^b^
non-HDL-C, mg/dl	141⋅1	5⋅2	146⋅8	5⋅7	0⋅465^b^
REM-C, mg/dl	8⋅0	1⋅3	6⋅1	1⋅8	0⋅112^c^
HDL-C, mg/dl	66⋅5	2⋅2	62⋅1	2⋅4	0⋅184^b^
TAG, mg/dl	100⋅1	5⋅7	114⋅6	10⋅8	0⋅301^c^
Glucose, mg/dl	98⋅2	1⋅4	101⋅4	1⋅9	0⋅679^c^
HbA1c, %	5⋅4	0⋅0	5⋅5	0⋅1	0⋅816^c^
Insulin, μU/ml	10⋅9	0⋅8	12⋅4	1⋅1	0⋅223^c^
hs-CRP, mg/dl	0⋅12	0⋅02	0⋅30	0⋅08	0⋅304^c^
Hcy, μmol/l	12⋅5	0⋅5	11⋅9	0⋅4	0⋅614^c^
Smoker status					
Never	43	60⋅6	27	49⋅1	0⋅238^a^
Ex	20	28⋅2	16	29⋅1	
Smoker	8	11⋅3	12	21⋅8	
Marital status					
Married	58	81⋅7	49	89⋅1	0⋅499^a^
Partner (unmarried)	4	5⋅6	1	1⋅8	
Single (not widowed)	6	8⋅5	2	3⋅6	
Single (widowed)	3	4⋅2	2	3⋅6	
Educational level					
Lower secondary school	15	21⋅1	22	40⋅0	**0⋅002**^a^
Secondary school	30	42⋅3	15	27⋅3	
University entrance qualification	11	15⋅5	15	27⋅3	
University degree	15	21⋅1	2	3⋅6	
PDI, points	29⋅0	1⋅8	25⋅4	2⋅3	0⋅205^b^
hPDI, points	–6⋅2	2⋅4	–19⋅1	2⋅3	**<0⋅001**^c^
uPDI, points	–35⋅6	2⋅5	–27⋅8	2⋅5	0⋅070^c^

Values are means ± sem except for qualitative variables which are expressed as *n* (%).

CCA, complete case analysis; ccIMT, common carotid intima-media thickness; BMI, body mass index; WC, waist circumference; BP, blood pressure; RHR, resting heart rate; TC, total cholesterol; LDL-C, LDL cholesterol; non-HDL-C, non-HDL cholesterol; REM-C, remnant cholesterol; HDL-C, HDL cholesterol; TAG, triacylglycerols; hs-CRP, high-sensitivity C-reactive protein; Hcy, homocysteine; PDI, plant-based diet index; hPDI, healthful PDI; uPDI, unhealthful PDI; sem, standard error of the mean; IN, intervention; CON: control.

*P*-values in bold are values below 0.05.

* *P*-value for between-group comparisons by:

a Fisher's exact test (two-sided).

b Independent *t* test (two-sided).

c Mann–Whitney *U* test (two-sided).

TC, measured LDL-C, non-HDL-C, REM-C, HDL-C, TAG, glucose, HbA1c, insulin: *n* 70 (IN); calculated LDL-C, PDI, hPDI, uPDI: *n* 70 (IN), *n* 54 (CON); hs-CRP: *n* 54 (IN), *n* 36 (CON); Hcy: *n* 57 (IN), *n* 54 (CON); Marital status, educational level: *n* 54 (CON).

Furthermore, a higher percentage of participants with a history of cancer (intervention: 9⋅9 %; control: 0 %; *P* = 0⋅018) and a higher percentage of participants with a family history (parents) of myocardial infarction or stroke (intervention: 56⋅3 %; control: 36⋅4 %; *P* = 0⋅031) were observed in the intervention group.

At baseline, there were no significant between-group differences in terms of ccIMT and a variety of other CVD parameters (Table 1). Similarly, there were no significant between-group differences in terms of alcohol intake frequency or the percentage of participants with (based on baseline values) hypertension, high values for total cholesterol (TC), LDL cholesterol (LDL-C; measured and calculated), non-HDL cholesterol (non-HDL-C), triacylglycerols (TAG), glucose or HbA1c or who had low HDL cholesterol (HDL-C). Furthermore, there were no significant between-group differences in terms of the percentage of those with a history of stroke, with a family history (siblings, grandparents) of myocardial infarction or stroke, or with any of a large variety of diagnosed disease conditions (hypertension, dyslipidaemia, heart disease, stroke, peripheral artery disease, diabetes, retinopathy, peripheral neuropathy, diabetic foot, kidney disease, allergies, gastrointestinal disease, thyroid disease, depression, rheumatoid arthritis, chronic pain, lung disease, bone disease as well as ‘other disease’ and ‘free of diagnosed disease’). There were no significant differences in any of the baseline characteristics (as listed in Table 1) between the group of participants with complete ccIMT data and with incomplete ccIMT data (*P* > 0⋅07).

### Seminar attendance

Compliance in the intervention group, defined as seminar attendance during the 10-week intensive phase of the lifestyle intervention, was relatively high: 60 out of the 71 evaluable participants (84⋅5 %) attended ≥10 (out of 14) seminars.

### Repeatability of ccIMT measurements

Repeatability (within-assay precision) of the two repeated measurements of mean and max ccIMT was good at all time points (mean ccIMT: *r* > 0⋅94; max ccIMT: *r* > 0⋅88). Mean differences in repeated measurements were small for both mean ccIMT (between 0⋅000 and 0⋅005 mm) and max ccIMT (between 0⋅003 and 0⋅007 mm).

### Mean ccIMT changes from baseline to 1 year

**Fig. 2.**
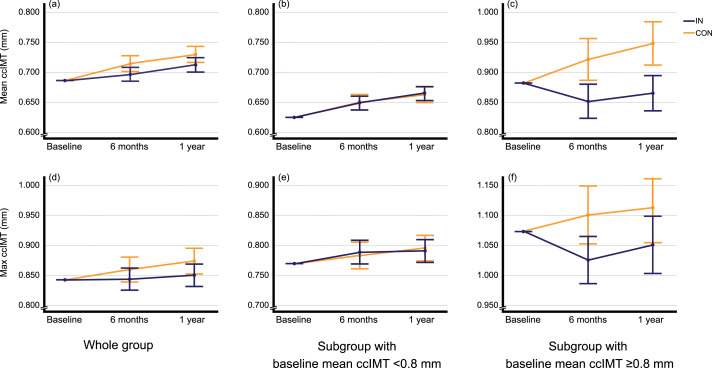
1-year mean and max ccIMT trajectories. Values are means and 95 % confidence intervals (adjusted for baseline). Whole group: (a and d) (IN: *n* 71; CON: *n* 55); subgroup with baseline mean ccIMT <0⋅8 mm: (b and e) (IN: *n* 53; CON: *n* 43); subgroup with baseline mean ccIMT ≥0⋅8 mm: (c and f) (IN: *n* 18; CON: *n* 12); *P*-values for between-group difference in 1-year trajectories of ccIMT (by ANCOVA; adjusted for baseline): (a) *P* = 0⋅022; (b) *P* = 0⋅970; (c) *P* < 0⋅001; (d) *P* = 0⋅117; (e) *P* = 0⋅965; (f) *P* = 0⋅023. ccIMT, common carotid intima-media thickness; IN, intervention group; CON, control group.

From baseline to 1 year, mean ccIMT significantly increased in both the intervention (0⋅026 [95 % CI 0⋅012, 0⋅039] mm; *P* = 0⋅001; *n* 71) and control group (0⋅045 [95 % CI 0⋅033, 0⋅056] mm; *P* < 0⋅001; *n* 55; Fig. 2; Supplementary Table S1). The 1-year trajectory of mean ccIMT was significantly lower in the intervention group compared to control (between-group difference: –0⋅012 [95 % CI –0⋅022, –0⋅002] mm; *P* = 0⋅022; adjusted for baseline mean ccIMT). This result remained significant after Holm–Bonferroni correction. This result was also confirmed when adjusting for baseline mean ccIMT, age and sex (*P* = 0⋅038; Fig. 2; Supplementary Table S1) or additionally for educational level, history of cancer, family history (parents) of myocardial infarction or stroke, and baseline hPDI (*P* = 0⋅045). Furthermore, this result was confirmed after adjusting (in addition to baseline mean ccIMT, age and sex) for baseline smoker status and alcohol intake (*P* = 0⋅040), for changes in smoker status and alcohol intake (*P* = 0⋅028), for baseline glucose, HbA1c, systolic BP and pulse pressure (*P* = 0⋅029), for baseline homocysteine (Hcy; *P* = 0⋅041) or for changes (Δ[baseline, 1 year]) in diastolic BP (*P* = 0⋅039). Apart from baseline mean ccIMT, none of the covariates had a significant influence on the models. Using log-transformed covariates confirmed the results.

However, in a sensitivity analysis comparing the 1½-year trajectories of mean ccIMT, the between-group difference was non-significant (–0⋅010 [95 % CI –0⋅022, 0⋅003] mm; *P* = 0⋅119; adjusted for baseline; intervention: *n* 63; control: *n* 47; Supplementary Table S2). Similarly, a sensitivity analysis with imputed data (LOCF) demonstrated no significant between-group difference in 1-year trajectories of mean ccIMT (*P* = 0⋅815; adjusted for baseline; intervention: *n* 101; control: *n* 75; Supplementary Table S3). A sensitivity analysis with imputed data (LOCF) comparing the 1½-year trajectories also attenuated the results (*P* = 0⋅853; adjusted for baseline). In addition, the favourable effect of the intervention was also attenuated in sensitivity analyses using log-transformed (lg10) mean ccIMT values (1-year trajectories: *P* = 0⋅051; 1½-year trajectories: *P* = 0⋅277; adjusted for baseline).

### Max ccIMT changes from baseline to 1 year

From baseline to 1 year, max ccIMT non-significantly increased in the intervention group (0⋅006 [95 % CI −0⋅015, 0⋅028] mm; *P* = 0⋅165; *n* 71) but significantly increased in the control group (0⋅034 [95 % CI 0⋅015, 0⋅053] mm; *P* = 0⋅001; *n* 55; Fig. 2; absolute values at each time point are can be found in Supplementary Table S1). The 1-year trajectory of max ccIMT was not significantly different between the intervention and control groups (between-group difference: −0⋅013 [95 % CI −0⋅030, 0⋅003] mm; *P* = 0⋅117; adjusted for baseline max ccIMT). This between-group difference remained non-significant after adjusting for baseline max ccIMT, age and sex (*P* = 0⋅098; Fig. 2; absolute values at each time point can be found in Supplementary Table S1). In contrast, the 1-year trajectory of max ccIMT was significantly lower after adjusting for baseline max ccIMT, age, sex, educational level, history of cancer, family history (parents) of myocardial infarction or stroke, and baseline hPDI (*P* = 0⋅041) or for baseline max ccIMT, age, sex and changes in smoker status and alcohol intake (*P* = 0⋅045). This result was again attenuated when adjusting (in addition to baseline max ccIMT, age and sex) for baseline smoker status and alcohol intake (*P* = 0⋅093), for baseline TAG, glucose, HbA1c, systolic BP, pulse pressure and waist circumference (*P* = 0⋅061), for baseline hs-CRP (*P* = 0⋅154), for baseline Hcy (*P* = 0⋅093) or for changes (Δ[baseline, 1 year]) in body weight and BMI (*P* = 0⋅164). Apart from baseline max ccIMT, none of the covariates had a significant influence on the models. Using log-transformed covariates confirmed the results.

Similarly, this result was confirmed by a sensitivity analysis comparing the 1½-year trajectories of max ccIMT (−0⋅014 [95 % CI −0⋅034, 0⋅006] mm; *P* = 0⋅164; adjusted for baseline; Supplementary Table S2), a sensitivity analysis with imputed data (LOCF; *P* = 0⋅756; adjusted for baseline; Supplementary Table S3) as well as a sensitivity analysis with imputed data (LOCF) comparing the 1½-year trajectories of max ccIMT (*P* = 0⋅846; adjusted for baseline). Similarly, this result was confirmed in sensitivity analyses using log-transformed (lg10) max ccIMT values (1-year trajectories: *P* = 0⋅200; 1½-year trajectories: *P* = 0⋅295; adjusted for baseline).

### Mean ccIMT changes from baseline to 1 year (subgroup analysis)

In a subgroup analysis including only participants with mean ccIMT ≥0⋅800 mm, from baseline to 1 year, mean ccIMT non-significantly decreased in the intervention group (−0⋅016 [95 % CI −0⋅050, 0⋅017] mm; *P* = 0⋅311; *n* 18) and significantly increased in the control group (0⋅065 [95 % CI 0⋅033, 0⋅096] mm; *P* = 0⋅001; *n* 12; Fig. 2; Supplementary Table S4). The 1-year trajectory of mean ccIMT was significantly lower in the intervention group than in control (between-group difference: −0⋅051 [95 % CI −0⋅075, −0⋅027] mm; *P* < 0⋅001; adjusted for baseline mean ccIMT). This result remained significant after adjusting for baseline mean ccIMT, sex and age (*P* < 0⋅001; Fig. 2; absolute values at each time point can be found in Supplementary Table S4). Due to the low number of cases in the subgroup, only these covariates were adjusted for. However, in sensitivity analyses, further covariates were added: the between-group difference remained significant after adjusting for baseline mean ccIMT, age, sex, educational level, history of cancer, family history (parents) of myocardial infarction or stroke and baseline hPDI (*P* < 0⋅001). Similarly, this result was confirmed when adjusting (in addition to baseline mean ccIMT, age and sex) for baseline smoker status and alcohol intake (*P* < 0⋅001), for changes in smoker status and alcohol intake (*P* < 0⋅001), for baseline glucose, HbA1c, systolic BP and pulse pressure (*P* < 0⋅001), for baseline Hcy (*P* < 0⋅001) or for changes (Δ[baseline, 1 year]) in diastolic BP (*P* < 0⋅001). Apart from baseline mean ccIMT, age and sex, none of the covariates had a significant influence on any of the models. Using log-transformed covariates confirmed the results.

Furthermore, this result was confirmed in a sensitivity analysis comparing the 1½-year trajectories (*P* = 0⋅001; adjusted for baseline; Supplementary Table S5), a sensitivity analysis with imputed data (LOCF) comparing the 1-year trajectories (*P* < 0⋅001; adjusted for baseline; intervention: *n* 23; control: *n* 15; Supplementary Table S6), and a sensitivity analysis with imputed data (LOCF) comparing the 1½-year trajectories (*P* = 0⋅002; adjusted for baseline; *P* < 0⋅001, adjusted for baseline, age and sex).

In addition, the favourable effect of the intervention was confirmed in sensitivity analyses using log-transformed (lg10) mean ccIMT values (1-year trajectories: *P* < 0⋅001; 1½-year trajectories: *P* = 0⋅001; adjusted for baseline). Furthermore, a sensitivity analysis with a cut-off value of 0⋅790 mm (the 75th percentile of baseline mean ccIMT in our study population) confirmed the results (intervention: *n* 19; control: *n* 12; between-group difference: −0⋅059 [95 % CI −0⋅082, −0⋅035] mm; *P* < 0⋅001; adjusted for age and sex).

### Max ccIMT changes from baseline to 1 year (subgroup analysis)

In the same subgroup, from baseline to 1 year, max ccIMT non-significantly decreased in the intervention group (−0⋅023 [95 % CI −0⋅071, 0⋅025] mm; *P* = 0⋅327; *n* 18) and non-significantly increased in the control group (0⋅041 [95 % CI −0⋅020, 0⋅102] mm; *P* = 0⋅168; *n* 12; Fig. 2; absolute values at each time point can be found in Supplementary Table S4). The 1-year trajectory of max ccIMT was significantly lower in the intervention group than in control (between-group difference: −0⋅046 [95 % CI −0⋅085, −0⋅007] mm; *P* = 0⋅023; adjusted for baseline max ccIMT). Adjusting for baseline max ccIMT, age and sex confirmed this result (*P* = 0⋅003). In sensitivity analyses, this result was confirmed when adjusting (in addition to baseline max ccIMT, age and sex) for educational level, history of cancer, family history (parents) of myocardial infarction or stroke and baseline hPDI (*P* = 0⋅007), for baseline smoker status and alcohol intake (*P* = 0⋅001), for changes in smoker status and alcohol intake (*P* = 0⋅004), for baseline TAG, glucose, HbA1c, systolic BP, pulse pressure and waist circumference (*P* = 0⋅003), for baseline hs-CRP (*P* = 0⋅027), for baseline Hcy (*P* = 0⋅008) or for changes (Δ[baseline, 1 year]) in body weight and BMI (*P* = 0⋅001). Apart from baseline max ccIMT, age and sex, none of these covariates had a significant influence on any of the models. Using log-transformed covariates confirmed the results.

A sensitivity analysis comparing the 1½-year trajectories of max ccIMT attenuated the result when adjusting for baseline max ccIMT (*P* = 0⋅061; adjusted for baseline; Supplementary Table S5) but confirmed the result when adjusting for baseline max ccIMT, age and sex (*P* = 0⋅011; Supplementary Table S5). This result was also confirmed by a sensitivity analysis with imputed data (LOCF) comparing the 1-year trajectories (*P* = 0⋅017; adjusted for baseline; Supplementary Table S6) and a sensitivity analysis with imputed data (LOCF) comparing the 1½-year trajectories of max ccIMT (*P* = 0⋅023, adjusted for baseline; *P* = 0⋅002, adjusted for baseline, age and sex).

In addition, the favourable effect of the intervention was also confirmed in sensitivity analyses using log-transformed (lg10) max ccIMT values (1-year trajectories: *P* = 0⋅004; 1½-year trajectories: *P* = 0⋅020; adjusted for baseline and age). Furthermore, a sensitivity analysis with a cut-off value of 0⋅790 mm (the 75th percentile of baseline mean ccIMT in our study population) confirmed the results (intervention: *n* 19; control: *n* 12; between-group difference: −0⋅065 [95 % CI −0⋅104, −0⋅026] mm; *P* = 0⋅002; adjusted for age and sex).

### Changes in diet scores and physical activity from baseline to 1 year

Compared to control, in the intervention group, the 1-year trajectories of PDI and hPDI were higher by 2⋅7 (95 % CI 1⋅7, 3⋅6) food portions/day and 3⋅9 (95 % CI 2⋅7, 5⋅0) food portions/day, respectively, while the 1-year trajectory of uPDI showed a decrease of −2⋅7 (95 % CI −3⋅7, −1⋅7) food portions/day (between-group differences: *P* < 0⋅001; adjusted for baseline). Sensitivity analyses confirmed that the favourable dietary changes were maintained at 1½ years (between-group differences: *P* < 0⋅001; adjusted for baseline).

A *post hoc* analysis showed that in the intervention group the 1-year trajectory of a modified hPDI (excluding the food groups potatoes, fish, eggs and dairy) was higher by 3⋅7 (95 % CI 2⋅7, 4⋅6) food portions/day (compared to control; between-group difference: *P* < 0⋅001; adjusted for baseline). These results were maintained at 1½ years (between-group differences: *P* < 0⋅001; adjusted for baseline).

At the food group level, results confirmed that participants of the intervention group were adhering to the recommendations, with significantly increased intakes (1-year trajectories) of fruit, vegetables, whole grains, legumes and nuts (approximately half a food portion/day higher, compared to control) as well as small increases in vegetable oil and fish intake. Conversely, in the intervention group, decreased intakes of meat, sweets/desserts and refined grains (approximately −0⋅3 to −0⋅4 portions/day) were observed, with small decreases also in the intakes of margarine, eggs and miscellaneous animal-source foods (compared to control). There were no significant between-group differences regarding the intakes (1-year trajectories) of tea, coffee, fruit juice, potatoes, sugar-sweetened beverages, animal fats, dairy or alcohol. No significant between-group differences were observed for the 1-year trajectories of intense, moderate or gentle physical activity (*P* > 0⋅09). This was confirmed for the 1½-year trajectories of physical activity (*P* > 0⋅10).

### Bivariate correlations of changes (Δ[baseline, 1 year]) in ccIMT and other markers

Bivariate correlations of changes in mean and max ccIMT with changes in other CVD markers as well as changes in dietary scores are shown in Table 2. For mean ccIMT change, a significant (although weak) correlation was only observed with changes in diastolic BP (Table 2). For max ccIMT change, significant correlations were only observed for changes in body weight, BMI and uPDI (Table 2). In addition, mean ccIMT change inversely correlated with baseline mean ccIMT (*r* = −0⋅179; *P* = 0⋅044), and max ccIMT change inversely correlated with baseline max ccIMT (*r* = −0⋅259; *P* = 0⋅003).

**Table 2.** Bivariate correlations of changes (Δ[baseline, 1 year]) in ccIMT and other markers

Parameter changes	Correlation with mean ccIMT change		Correlation with max ccIMT change		*n*
	Correlation coefficient	*P*-value	Correlation coefficient	*P*-value	
Body weight	0⋅165	0⋅066	0⋅207	**0⋅020**	125
BMI	0⋅169	0⋅059	0⋅206	**0⋅021**	125
WC	0⋅077	0⋅393	0⋅144	0⋅109	125
Systolic BP	0⋅169	0⋅059	0⋅114	0⋅204	125
Diastolic BP	0⋅182	**0⋅042**	0⋅057	0⋅531	125
Pulse pressure	0⋅051	0⋅571	0⋅117	0⋅193	125
RHR	−0⋅065	0⋅472	−0⋅044	0⋅629	125
TC	−0⋅048	0⋅599	0⋅012	0⋅893	124
LDL-C (measured)	−0⋅060	0⋅505	−0⋅009	0⋅922	124
LDL-C (calculated)	−0⋅066	0⋅470	−0⋅005	0⋅959	123
Non-HDL-C	−0⋅074	0⋅415	−0⋅013	0⋅882	124
REM-C	0⋅015	0⋅865	0⋅033	0⋅715	124
HDL-C	−0⋅006	0⋅948	0⋅008	0⋅934	124
TAG	−0⋅006	0⋅947	0⋅007	0⋅941	124
Glucose	−0⋅019	0⋅833	0⋅074	0⋅417	124
HbA1c	0⋅032	0⋅728	0⋅116	0⋅199	124
Insulin	−0⋅109	0⋅227	−0⋅019	0⋅831	124
hs-CRP	−0⋅062	0⋅541	0⋅013	0⋅901	98
Hcy	−0⋅084	0⋅384	−0⋅140	0⋅144	110
PDI	0⋅103	0⋅262	−−0⋅031	0⋅741	120
hPDI	−0⋅043	0⋅638	−0⋅145	0⋅113	120
uPDI	0⋅075	0⋅413	0⋅240	**0⋅008**	120

Correlations coefficients: spearman's rho; ccIMT, common carotid intima-media thickness; BMI, body mass index; WC, waist circumference; BP, blood pressure; RHR, resting heart rate; TC, total cholesterol; LDL-C, LDL cholesterol; non-HDL-C, non-HDL cholesterol; REM-C, remnant cholesterol; HDL-C, HDL cholesterol; TAG, triacylglycerols; hs-CRP, high-sensitivity C-reactive protein; Hcy, homocysteine; PDI, plant-based diet index; hPDI, healthful PDI; uPDI, unhealthful PDI.

*P*-values in bold are values below 0.05.

In the subgroup of participants with baseline mean ccIMT ≥0⋅800 mm, changes in uPDI positively correlated with changes in mean ccIMT (*r* = 0⋅370; *P* = 0⋅048) and max ccIMT (*r* = 0⋅539; *P* = 0⋅003) [*n* 29], while no significant correlations between changes in mean or max ccIMT and changes in PDI, hPDI or CVD markers were observed.

A *post hoc* analysis showed that changes in the modified hPDI (excluding the food groups potatoes, fish, eggs and dairy) inversely correlated with max ccIMT change (*r* = −0⋅229; *P* = 0⋅012). This result was confirmed in the subgroup of participants with high baseline ccIMT (*r* = −0⋅460; *P* = 0⋅012). Mean ccIMT change did not significantly correlate with changes in modified hPDI.

### Mean and max ccIMT changes stratified by baseline risk factors

1-year trajectories of mean ccIMT and max ccIMT did not significantly differ between men and women (*P* = 0⋅313; *P* = 0⋅208) or between participants with or without overweight (*P* = 0⋅365; *P* = 0⋅707) or with or without obesity (*P* = 0⋅940; *P* = 0⋅938). Similarly, 1-year trajectories of mean ccIMT and max ccIMT did not significantly differ by smoker status (*P* = 0⋅467; *P* = 0⋅449), categories of alcohol intake (*P* = 0⋅547; *P* = 0⋅591), marital status (*P* = 0⋅921; *P* = 0⋅931) or educational level (*P* = 0⋅578; *P* = 0⋅579; all adjusted for baseline).

## Discussion

The present study had the aim of examining the effect of a community-based lifestyle intervention on changes in mean and max ccIMT (among other CVD markers^(16)^). While the intervention resulted in significant dietary improvements, in line with the recommendations given, physical activity did not significantly increase over the course of 1 year. Previously we reported that, with all participants included, the intervention had no significant effect on ccIMT within 6 months in the whole study group but that in a subgroup of participants with high baseline ccIMT (mean ccIMT ≥0⋅800 mm) the intervention resulted in significantly lower (more favourable) mean and max ccIMT changes compared to control^(17)^. The present 1-year analyses (and 1½-year sensitivity analyses) confirm this result of significantly slowed down mean ccIMT and max ccIMT progression in the subgroup with high baseline ccIMT. This result is in line with the results of the PREDIMED-Navarra study, a 1-year controlled intervention with a traditional Mediterranean diet, which showed significant effects on ccIMT only in the subgroup with high baseline ccIMT (mean ccIMT ≥0⋅9 mm)^(21)^. Comparable subgroup results from other studies seem to not have been published^(22)^.

A recent meta-analysis suggests that every 0⋅010 mm reduction in mean ccIMT progression can reduce CVD event risk by 10–15 %^(12)^. Therefore, the between-group difference (−0⋅051 [95 % CI −0⋅075, −0⋅027] mm) observed in the high-baseline subgroup of the present study appears to be clinically relevant. In contrast to the previously reported 6-month results^(17)^, with all participants included, the present 1-year analysis showed a significantly lower (slowed down) mean ccIMT progression in the intervention group. However, this between-group difference (−0⋅012 [95 % CI −0⋅022, −0⋅002] mm) was small and was attenuated in sensitivity analyses comparing the 1½-year trajectories. Nevertheless, it can be hypothesised that the clinical benefit of this effect may increase over time, if participants maintain the achieved lifestyle changes in the future^(20,23)^.

To date, only a small number of controlled trials have assessed the effect of a combined lifestyle intervention (including diet and other lifestyle factors) on ccIMT^(22,24–28)^, and only two of these trials were conducted with generally healthy participants^(27,28)^: in a suburban population in Japan, Okada *et al.* observed no significant effect of a 2-year lifestyle modification on mean ccIMT change (compared to control; dietary recommendations were based on the goals of the National Cholesterol Education Program; <30 % total fat, <7 % saturated fat and <200 mg dietary cholesterol per day)^(27)^. Similarly, in a study with perimenopausal women in the United States, Wildman *et al.* observed no significant effect of a 4-year diet (≤25 % total fat, ≤7 % saturated fat, ≤100 mg dietary cholesterol and 1300 kcal/d) and exercise intervention on mean ccIMT change (compared to control)^(28)^. In terms of diet-only interventions (i.e. interventions focused solely on a change in dietary pattern), no controlled trials assessing ccIMT change seem to have been conducted with generally healthy participants. Thus, our study appears to be the first controlled trial (lifestyle or dietary) to have shown a significant intervention effect on ccIMT in a community-based sample of mostly generally healthy participants from the general population.

Apart from our study, only one controlled trial appears to have assessed the effect of a combined lifestyle intervention including a strong focus on a predominantly plant-based diet on ccIMT^(25)^. In the present study, Aldana *et al.* observed no effect of a 1-year intervention including a very low-fat plant-based diet (Ornish Program) on ccIMT in patients with clinically confirmed coronary artery disease (compared to usual care)^(25)^. In contrast to our study, healthful plant-based high-fat foods (such as nuts, extra virgin olive oil or cold-pressed rapeseed oil) were not recommended in the Ornish Program^(25)^, whereas more recent evidence suggests that these foods may not just improve cardiovascular health in general^(29)^ but may also beneficially affect ccIMT^(20,21)^. In terms of diet-only interventions, five controlled trials have assessed the effect of moving towards a more plant-based diet on ccIMT: four of these studies used a Mediterranean diet (in Spain^(20,21,30)^ and Italy^(23)^), and one study used a Mediterranean-like diet (in Norway^(31,32)^). All of these studies were conducted with participants at higher CVD risk, with four out of these five studies showing a significant favourable effect on ccIMT^(20,21,23,31,32)^. In contrast, two controlled trials observed that a low-carbohydrate diet had no significant effect on ccIMT in type 2 diabetes patients after 1 year (United States^(33)^) and 1½ years (Taiwan^(34)^), respectively. As no comparable studies could be identified, our study appears to be the first controlled trial (lifestyle or dietary) which tested the effect of recommending a predominantly plant-based diet (centred around fruit, vegetables, whole grains, legumes, nuts/seeds and healthy oils) on ccIMT in a study sample of mostly generally healthy participants^(31)^.

Similarly, our study appears to be the first controlled trial (lifestyle or dietary) to assess correlations between ccIMT changes and changes in PDI, hPDI and uPDI as well as between ccIMT changes and Hcy changes. Apart from a dietary intervention study by Petersen *et al.*^(35)^, our study also appears to be the only controlled trial (lifestyle or dietary) to have correlated ccIMT changes with hs-CRP changes. While we observed that uPDI change positively correlated with max ccIMT change (and in the subgroup analyses with both mean and max ccIMT changes), we did not observe significant correlations of ccIMT change with changes in Hcy or hs-CRP. As in our study, Petersen *et al.* found no significant correlation of mean ccIMT change with hs-CRP change^(35)^. The positive correlations of uPDI change with changes in mean ccIMT (*r* = 0⋅370) and max ccIMT (*r* = 0⋅539), which we observed in the high-baseline subgroup, indicate that the favourable effects on ccIMT were partially mediated by the observed decrease in uPDI. The results do not indicate a strong role of PDI or hPDI changes in the observed favourable effects. However, the significant inverse correlation of a modified hPDI (excluding the food groups potatoes, fish, eggs and dairy) with max ccIMT change which we observed indicates that an increased intake of healthful plant foods and a decreased intake of certain animal-source foods may also contribute to lower max ccIMT progression, although we observed a significant beneficial effect on max ccIMT change only in the subgroup analysis.

Among diet-only interventions, Jimenez *et al.* and Maiorino *et al.* observed a favourable effect on mean ccIMT in their intervention groups following a traditional Mediterranean diet after 5 and 7 years (coronary heart disease patients; Spain^(20)^) and after 4 and ~8 years (type 2 diabetes patients; Italy^(23)^), respectively. It should be noted that both studies compared their intervention groups to control groups following healthy low-fat diets^(20,23)^, which may underestimate^(36)^ but may also overestimate the effect (e.g. Jimenez *et al*. observed a decrease in Mediterranean diet adherence in the low-fat control^(20)^). In addition to the significant effects at 4 and ~8 years, Maiorino *et al.* observed a non-significant, favourable effect on mean ccIMT change at 2 years (between-group difference: *P* = 0⋅050^(23)^), which indicates that longer study durations may increase the likelihood of observing significant effects on ccIMT. While in our study dietary improvements (compared to control) were similarly maintained at 1 year and 1½ years, it should be considered that in lifestyle and dietary interventions, adherence to recommendations may decrease over time, especially in generally healthy participants, and that dietary changes may occur in the no-intervention control group. Furthermore, our study indicates that dropout rates can be high in study populations of generally healthy individuals, potentially making long-term (≥4 years)^(23)^ follow-up less feasible. We observed that the small but significant intervention effect on mean ccIMT at 1 year (*P* = 0⋅022) became non-significant at 1½ years (*P* = 0⋅119), which may have been influenced by the lower number of participants available at 1½ years. In their study with type 1 and type 2 diabetes patients in Australia, Petersen *et al.* were able to demonstrate a significant favourable effect of repeated counselling from a dietitian on mean and max ccIMT after 1 year (compared to usual diet)^(35)^. Petersen *et al.* observed that mean ccIMT change was ~0⋅016 mm lower in the intervention group (compared to control)^(35)^, whereas our study found that mean ccIMT was lower by 0⋅012 mm in the intervention group. While the dietary instructions given to the intervention group by Petersen *et al.* were to increase the intakes of fruit (not fruit juice), vegetables and dairy (milk or yoghurt, not cheese) and at 3 months increased intakes of fruit (+179 g/d), vegetables (+46 g/d) and yoghurt (+38 g/d) were observed (compared to control), these increases were not maintained at 1 year^(35)^. In comparison, we observed increased intakes (1-year trajectories) of about half a food portion/day for fruit, vegetables, whole grains and legumes (equivalent to an increase of about 50–75 g/d each) as well as additional dietary changes (including a reduction in the intakes of meat, refined grains and sweets) in the intervention group (compared to control).

Our subgroup analyses confirm that significant effects on ccIMT may be more easily demonstrated in individuals with higher baseline ccIMT^(21)^, and such higher ccIMT values are more likely to be present in study samples of individuals with confirmed CVD risk factors such as hypercholesterolaemia^(31)^ or diabetes^(23,35)^. Nevertheless, it is of high public health relevance to develop tools (such as our intervention programme) which already initiate CVD prevention measures in individuals who are still at low to moderate CVD risk^(37)^. Consequently, it appears justifiable to conduct further lifestyle/dietary interventions assessing ccIMT change in generally healthy participants. It should also be considered that with participants at higher CVD risk a control group for which no effect (no intervention) or a lower effect can be expected may be ethically problematic^(38)^. Like Petersen *et al.*^(35)^ (and no other controlled lifestyle/dietary trial), our study showed a significant effect on mean ccIMT change after 1 year. At a follow-up of <1 year, significant favourable effects of lifestyle/dietary pattern modification on ccIMT change (compared to control) have only been shown in type 2 diabetes patients (after 6 months; South Korea)^(26)^ as well as in our subgroup analysis at 6 months^(17)^.

A cut-off value for mean ccIMT of 0⋅8 mm, as used in our study, has been widely utilised as a threshold for describing what constitutes elevated mean ccIMT values^(39)^. It has furthermore been proposed that the age-, sex- and race-specific 75th percentile of mean ccIMT (derived from large cohort studies) should be used as a cut-off value^(1,40)^. In our study population (evaluable participants; *n* 126), the 75th percentile of baseline mean ccIMT was 0⋅786 mm, which confirms the usefulness of 0⋅800 mm as a cut-off value in our study. Sensitivity analyses with a cut-off value of 0⋅790 mm confirmed the results.

Only a small number of controlled trials have tested the effect of exercise-only interventions on ccIMT^(41–46)^. One small study demonstrated a significant favourable effect of exercise training on ccIMT (compared to control)^(41,43)^, and one other study observed a significant beneficial effect only in a subgroup analysis of patients without identified carotid plaques^(42)^. The other studies did not demonstrate significant effects compared to control.

Age-related ccIMT increase is partly mediated by increased sympathetic nerve activity in vascular smooth muscle^(47)^. Psychological stress is associated with increased sympathetic activity^(48)^, blood pressure^(49)^ and ccIMT^(50)^, and this highlights the importance of psychological stress management as a component of healthy lifestyle recommendations^(51,52)^. In the present study, we observed a significant reduction in body weight in the intervention group (compared to control). Weight loss is associated with decreased sympathetic activity^(53)^ and decreased ccIMT^(54)^, and in the present study a positive correlation was observed between changes in body weight and changes in max ccIMT (*P* = 0⋅020) and mean ccIMT (*P* = 0⋅066). However, in the high-baseline subgroup, changes in body weight did not significantly correlate with changes in mean ccIMT (*P* = 0⋅387) or max ccIMT (*P* = 0⋅640). Similarly, adjusting for body weight or BMI changes did not significantly influence the ANCOVA models (*P* > 0⋅69; unpublished results). This indicates that body weight reduction was not a main driver of the favourable effects on ccIMT which were observed in the subgroup.

### Strengths and limitations

A strength of the present study is the use of a no-intervention control group, a strict standardised measurement protocol^(1,19,55)^, several follow-up time points, high repeatability^(39)^ and having all ccIMT measurements conducted by the same technician and with the same device^(1,40)^.

Two relevant limitations are the non-randomised design and that the intervention study arm started 6 months earlier than the control group (same follow-up durations). While our findings in the subgroup with high baseline ccIMT seem robust, residual confounding may have remained, particularly since our trial was non-randomised and, as a community-based study, the study sample was non-homogeneous^(12,56)^. However, baseline characteristics indicate that both study groups were comparable. Furthermore, seasonal changes may have influenced the results, for example by way of improved vitamin D status during the summer^(57)^. However, in the subgroup, results of the 6-month^(17)^, 1-year and 1½-year analyses consistently demonstrated lower mean and max ccIMT trajectories in the intervention group. This indicates that these results are not strongly confounded by seasonal effects. A further limitation is that the relatively small sample size did not allow for a complex statistical analysis of all potential confounders.

## Conclusion

The results indicate that healthy lifestyle changes, as they were addressed in the Healthy Lifestyle Community Programme (cohort 2), can effectively reduce mean and max ccIMT if baseline ccIMT is above a value of 0⋅800 mm, indicating an elevated risk. The observed favourable effect of the intervention in participants with high baseline ccIMT likely constitutes a true deceleration of mean and max ccIMT progression. These results appear to be robust and are likely applicable to similar populations. Although in the present study most participants did not have elevated baseline ccIMT values, we still observed a significant and relevant beneficial intervention effect on ccIMT in the analysis including all participants. While the clinical benefit of the observed effect is likely greater in those with elevated ccIMT, it is equally an advantage to maintain normal ccIMT values within the normal range for as long as possible, if one takes ccIMT as a predictor of CVD risk. In the subgroup of individuals with low baseline ccIMT (<0⋅800 mm), lifestyle changes alone may not be sufficient or the lifestyle changes observed in the present study may not have been substantial enough to significantly improve ccIMT in the short term. As mean and max ccIMT are secondary end points, the results should be considered exploratory.

## Supplementary material

For supplementary material accompanying this paper visit https://doi.org/10.1017/jns.2022.46. click here to view supplementary material
