# Supplementary material for: Healthy lifestyle changes favourably affect common carotid intima-media thickness: the Healthy Lifestyle Community Programme (cohort 2)
Source: J Nutr Sci. 2022 Jun 13;11:e47. doi: 10.1017/jns.2022.46 (PMC9201878; doi:10.1017/jns.2022.46)
Supplement: Supplementary file 1 [file S2048679022000465sup001.pdf]

Supplementary material

Additional information on the ccIMT measurement protocol

All assessments were made by the same technician and with the same ultrasound device (Mindray DC-N3, Mindray, Shenzhen, China). Two ccIMT measurements were taken on each side, resulting in four mean ccIMT and four maximal (max) ccIMT values (per person, per measurement time point. Each participant was examined in the supine position, with their head at a 45° angle turned away from the examiner. The left and right common carotid arteries were scanned longitudinally, scanning a 1 cm-long, plaque-free artery section, located 1 cm proximal to the carotid bulb. Plaque was defined as a focal thickening in the intima-media complex of >1.5 mm. Measurements were taken with the artery displayed clearly horizontally on the screen and with the carotid bifurcation visible in the image as a landmark. All measurements were made in B-mode and greyscale (the standard method of measurement).

Supplementary table 1

Supplementary table 1. Mean and max ccIMT changes within 1 year (CCA)

**Parameters**	**Mean ccIMT, mm**				**Max ccIMT, mm**			
**Group**	**IN** (n = 71)		**CON** (n = 55)		**IN** (n = 71)		**CON** (n = 55)	
	Mean	SEM or 95% CI	Mean	SEM or 95% CI	Mean	SEM or 95% CI	Mean	SEM or 95% CI
**Baseline**	0.695	0.016	0.674	0.019	0.856	0.019	0.825	0.023
**6 months**	0.704	0.014	0.704	0.019	0.855	0.016	0.846	0.023
**1 year**	0.721	0.015	0.718	0.020	0.862	0.018	0.859	0.025
**Δ(baseline, 1 year)**	0.026	0.012, 0.039	0.045	0.033, 0.056	0.006	-0.015, 0.028	0.034	0.015, 0.053
**p WG ***	**0.001** a		**<0.001** a		0.165 a		**0.001** a	
**p BG †**	**0.022** b				0.117 b			
**p BG † (multivariable-adjusted)**	**0.038** c				0.098 c			

Values are means and SEM, except changes which are expressed as means and 95% CI.
ccIMT: common carotid intima-media thickness; CCA: complete case analysis; IN: intervention; CON: control; p WG: p-values for within-group changes from baseline to 1 year; p BG: p-values for between-group differences in 1-year trajectories; SEM: standard error of the mean; CI: confidence interval;
* p-value for within-group comparisons by:
[a] Wilcoxon test (two-sided)
† p-value for between-group comparisons by:
[b] repeated measures ANCOVA, adjusted for the baseline values
[c] repeated measures ANCOVA, adjusted for the baseline values, age, and sex

Supplementary table 2

**Supplementary table 2.** Mean and max ccIMT changes within 1½ years (CCA)								
**Parameters**	**Mean ccIMT, mm**				**Max ccIMT, mm**			
**Group**	**IN** (n = 63)		**CON** (n = 47)		**IN** (n = 63)		**CON** (n = 47)	
	Mean	SEM or 95% CI	Mean	SEM or 95% CI	Mean	SEM or 95% CI	Mean	SEM or 95% CI
**Baseline**	0.692	0.017	0.685	0.020	0.853	0.020	0.838	0.026
**6 months**	0.704	0.014	0.712	0.021	0.854	0.017	0.855	0.026
**1 year**	0.717	0.015	0.729	0.023	0.858	0.018	0.871	0.028
**1½ years**	0.723	0.015	0.722	0.022	0.862	0.017	0.869	0.027
**Δ(baseline, 1½ years)**	0.031	0.014, 0.048	0.037	0.025, 0.050	0.009	-0.017, 0.036	0.031	0.011, 0.051
**p WG ***	**<0.001** a		**<0.001** a		0.077 a		**0.004** a	
**p BG †**	0.119 b				0.164 b			
**p BG † (multivariable-adjusted)**	0.217 c				0.166 c			

Values are means and SEM, except changes which are expressed as means and 95% CI.
ccIMT: common carotid intima-media thickness; CCA: complete case analysis; IN: intervention; CON: control; p WG: p-values for within-group changes from baseline to 1½ years; p BG: p-values for between-group differences in 1½-year trajectories; SEM: standard error of the mean; CI: confidence interval;
* p-value for within-group comparisons by:
[a] Wilcoxon test (two-sided)
† p-value for between-group comparisons by:
[b] repeated measures ANCOVA, adjusted for baseline values
[c] repeated measures ANCOVA, adjusted for baseline values, age, and sex

Supplementary table 3

**Supplementary table 3.** Mean and max ccIMT changes within 1 year (LOCF)								
**Parameters**	**Mean ccIMT, mm**				**Max ccIMT, mm**			
**Group**	**IN** (n = 101)		**CON** (n = 75)		**IN** (n = 101)		**CON** (n = 75)	
	Mean	SEM or 95% CI	Mean	SEM or 95% CI	Mean	SEM or 95% CI	Mean	SEM or 95% CI
**Baseline**	0.688	0.013	0.671	0.016	0.851	0.016	0.820	0.020
**6 months**	0.702	0.012	0.691	0.017	0.856	0.015	0.831	0.021
**1 year**	0.719	0.012	0.702	0.018	0.865	0.016	0.842	0.021
**Δ(baseline, 1 year)**	0.031	0.018, 0.043	0.031	0.021, 0.042	0.014	-0.005, 0.033	0.022	0.006, 0.038
**p WG ***	**<0.001** a		**<0.001** a		**0.041** a		**0.003** a	
**p BG †**	0.815 b				0.756 b			
**p BG † (multivariable-adjusted)**	0.497 c				0.354 c			

Values are means and SEM, except changes which are expressed as means and 95% CI.
ccIMT: common carotid intima-media thickness; LOCF: last observation carried forward (imputed data); IN: intervention; CON: control; p WG: p-values for within-group changes from baseline to 1 year; p BG: p-values for between-group differences in 1-year trajectories; SEM: standard error of the mean; CI: confidence interval;
* p-value for within-group comparisons by:
[a] Wilcoxon test (two-sided)
† p-value for between-group comparisons by:
[b] repeated measures ANCOVA, adjusted for the baseline values
[c] repeated measures ANCOVA, adjusted for the baseline values, age, and sex

Supplementary table 4

Supplementary table 4. Subgroup analysis: mean and max ccIMT changes within 1 year (CCA)								
**Parameters**	**Mean ccIMT, mm**				**Max ccIMT, mm**			
**Group**	**IN** (n = 18)		**CON** (n = 12)		**IN** (n = 18)		**CON** (n = 12)	
	Mean	SEM or 95% CI	Mean	SEM or 95% CI	Mean	SEM or 95% CI	Mean	SEM or 95% CI
**Baseline**	0.886	0.015	0.876	0.023	1.062	0.018	1.089	0.028
**6 months**	0.855	0.017	0.917	0.025	1.018	0.019	1.112	0.035
**1 year**	0.870	0.023	0.941	0.031	1.039	0.029	1.130	0.042
**Δ(baseline, 1 year)**	-0.016	-0.050, 0.017	0.065	0.033, 0.096	-0.023	-0.071, 0.025	0.041	-0.020, 0.102
**p WG ***	0.311 a		**0.001** a		0.327 a		0.168 a	
**p BG †**	**<0.001** b				**0.023** b			
**p BG † (multivariable-adjusted)**	**<0.001** c				**0.003** c			

Values are means and SEM, except changes which are expressed as means and 95% CI. Subgroup: participants with baseline mean ccIMT ≥0.800 mm.
ccIMT: common carotid intima-media thickness; CCA: complete case analysis; IN: intervention; CON: control; p WG: p-values for within-group changes from baseline to 1 year; p BG: p-values for between-group differences in 1-year trajectories; SEM: standard error of the mean; CI: confidence interval;
* p-value for within-group comparisons by:
[a] paired t-test (two-sided)
† p-value for between-group comparisons by:
[b] repeated measures ANCOVA, adjusted for baseline values
[c] repeated measures ANCOVA, adjusted for baseline values, age, and sex

Supplementary table 5

Supplementary table 5. Subgroup analysis: mean and max ccIMT changes within 1½ years (CCA)								
**Parameters**	**Mean ccIMT, mm**				**Max ccIMT, mm**			
**Group**	**IN** (n = 15)		**CON** (n = 11)		**IN** (n = 15)		**CON** (n = 11)	
	Mean	SEM or 95% CI	Mean	SEM or 95% CI	Mean	SEM or 95% CI	Mean	SEM or 95% CI
**Baseline**	0.883	0.016	0.883	0.024	1.057	0.020	1.103	0.026
**6 months**	0.857	0.019	0.924	0.026	1.020	0.021	1.123	0.037
**1 year**	0.860	0.023	0.948	0.033	1.033	0.031	1.142	0.044
**1½ years**	0.876	0.020	0.937	0.034	1.042	0.022	1.134	0.046
**Δ(baseline, 1½ years)**	-0.007	-0.055, 0.041	0.054	0.028, 0.080	-0.016	-0.079, 0.048	0.031	-0.022, 0.084
**p WG ***	0.757 a		**0.001** a		0.610 a		0.221 a	
**p BG †**	**0.001** b				0.061 b			
**p BG † (multivariable-adjusted)**	**0.001** c				**0.011** c			

Values are means and SEM, except changes which are expressed as means and 95% CI. Subgroup: participants with baseline mean ccIMT ≥0.800 mm.
ccIMT: common carotid intima-media thickness; CCA: complete case analysis; IN: intervention; CON: control; p WG: p-values for within-group changes from baseline to 1½ years; p BG: p-values for between-group differences in 1½-year trajectories; SEM: standard error of the mean; CI: confidence interval;
* p-value for within-group comparisons by:
[a] paired t-test (two-sided)
† p-value for between-group comparisons by:
[b] Repeated measures ANCOVA, adjusted for the baseline values
[c] Repeated measures ANCOVA, adjusted for the baseline values, age, and sex

Supplementary table 6

**Supplementary table 6.** Subgroup analysis: mean and max ccIMT changes within 1 year (LOCF)								
**Parameters**	**Mean ccIMT, mm**				**Max ccIMT, mm**			
**Group**	**IN** (n = 23)		**CON** (n = 15)		**IN** (n = 23)		**CON** (n = 15)	
	Mean	SEM or 95% CI	Mean	SEM or 95% CI	Mean	SEM or 95% CI	Mean	SEM or 95% CI
**Baseline**	0.883	0.014	0.883	0.019	1.061	0.016	1.089	0.022
**6 months**	0.861	0.014	0.918	0.020	1.029	0.016	1.111	0.028
**1 year**	0.873	0.018	0.937	0.025	1.043	0.023	1.125	0.033
**Δ(baseline, 1 year)**	-0.010	-0.038, 0.019	0.054	0.027, 0.082	-0.018	-0.056, 0.020	0.036	-0.012, 0.084
**p WG ***	0.429 a		**0.002** a		0.340 b		0.128 b	
**p BG †**	**<0.001** c				**0.017** c			
**p BG † (multivariable-adjusted)**	**<0.001** d				**0.002** d			

Values are means and SEM, except changes which are expressed as means and 95% CI. Subgroup: participants with baseline mean ccIMT ≥0.800 mm.
ccIMT: common carotid intima-media thickness; LOCF: last observation carried forward (imputed data); IN: intervention; CON: control; p WG: p-values for within-group changes from baseline to 1 year; p BG: p-values for between-group differences in 1-year trajectories; SEM: standard error of the mean; CI: confidence interval;
* p-value for within-group comparisons by:
[a] Wilcoxon test (two-sided)
[b] paired t-test (two-sided)
† p-value for between-group comparisons by:
[c] Repeated measures ANCOVA, adjusted for the baseline values
[d] Repeated measures ANCOVA, adjusted for the baseline values, age, and sex

Additional information regarding the lifestyle recommendations

The healthy lifestyle handbook which the participants of the intervention group received contained chapters on the following topics: (1) opportunities and limitations of modern medicine, (2) a healthy lifestyle: the best medicine, (3) eat yourself healthy, (4) fibre: your good friend, (5) with fork and knife against overweight and type 2 diabetes, (6) concerns of the heart: fat & cholesterol, (7) blood pressure: the silent threat, (8) taking cancer prevention into your own hands, (9) physical activity: running away from illness, (10) manage stress – a life with more relaxation, (11) healthy and fit at any age, (12) an experiment: the contract between yourself and your health, (13) change is possible, (14) on the tracks of your personal strengths, (15) from "I" to "we".

The recipe booklet contained only vegetarian recipes (most of which were vegan) based on whole grains, legumes, vegetables, fruit, nuts/seeds, and healthy oils. While the recommendation to adopt an entirely vegetarian/vegan diet was not stated, the idea was to improve the typical German diet rich in animal-source foods and highly processed foods by adding more healthy plant foods.

An English translation of the laminated information sheet can be found at the end of this Supplementary material document (the following two pages).

Keep your diet in check!

Portion check: vegetables 1 ☐ 2 ☐ 3 ☐ 4 ☐ 5 ☐

Portion check: fruit 1 ☐ 2 ☐ 3 ☐ 4 ☐ 5 ☐

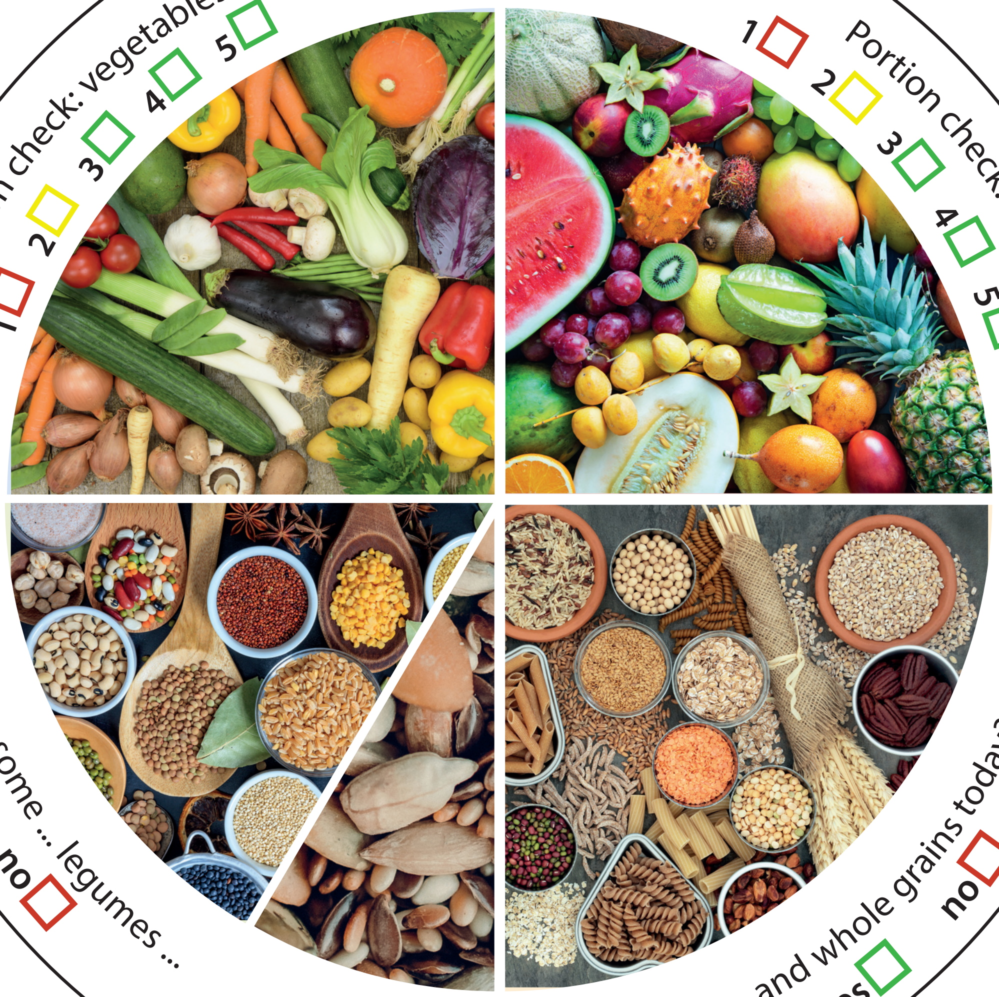

Did you have some ... legumes ... yes ☐ no ☐

... nuts/seeds ... yes ☐ no ☐

... and whole grains today? yes ☐ no ☐

Keep your habits in check!

Water

Today I had ...

1 ☐ 2 ☐ 3 ☐ 4 ☐ 5 ☐

6 ☐ 7 ☐ 8 ☐ 9 ☐ 10 ☐

... glasses of water or unsweetened tea!

Activity

I've done this today:

☐ unfortunately didn't exercise

☐ 30 min or 5 000 steps

☐ 60 min or 10 000 steps

Mindfulness

This many times today I took a minute off just for myself:

1 ☐ 2 ☐ 3 ☐ 4 ☐ 5 ☐ 6 ☐ 7 ☐ 8 ☐ 9 ☐ 10 ☐

Today I did something to make myself feel relaxed! yes ☐ no ☐

Satiety

Today after dinner I felt ...

☐ much too full

☐ a little too full

☐ comfortably full

Social life

☐ I received some support from others today.

☐ I took some time today to spend with friends or family.

☐ I did some socializing today.

Start every day with a kick-start!

Have more of these …

Fruit and vegetables
Eat at least 2 portions of fresh fruit a day. Avoid canned fruit, and use dried fruit for sweetening. Enjoy a variety of different types of vegetables and eat 3 or more portions every day.

Legumes and potatoes
Enjoy legumes such as lentils, soya foods (for example, tofu), peas, and beans daily. Eat boiled or baked potatoes or sweet potatoes and avoid high-calorie sauces.

Whole grains and whole grain products
Whole grain cereals such as millet, barley, quinoa, rolled oats and wheat can be eaten in breakfast porridge or as side or main dishes. Also enjoy bread, rice, pasta, etc. in their whole-grain varieties.

Nuts, seeds, and oils
Preferably consume rapeseed and olive oil. Make nuts and seeds (unsalted!) a regular part of your diet. But don‘t eat more than a handful a day.

Water
Drink 10 glasses of water or unsweetened tea every day. Spice up your water with some fresh herbs, slices of lemon or ginger, or pieces of fresh fruit.

Have less of these …

Animal fats and animal protein
Eat meat or offal 0 to 2 times a week. Avoid sausages and processed meats if possible.
Use low-fat milk and dairy products, and try out plant-based alternatives.

Sugar, sweets, and snacks
Consume sugar, honey, syrup, cakes, sweets, soft drinks, high-sugar desserts and spread in small quantities only. And save these for special occasions.

Salt
Use salt sparingly. Discover the large variety of fresh herbs and spices for seasoning your meals!
Do not consume salty foods such as crisps, salted nuts, crackers, soya sauce, or convenience foods (for example, pizza) or consume them in moderation.

Eggs
Eat 0 to 3 eggs a week. Also watch out for hidden eggs in cakes pasta, and cookies.

Alcohol
Do not drink alcohol more than once or twice a week. Reserve drinking alcohol for special occasions and have only small amounts.

Establish healthy habits!

Food habits

- ✓ After getting up, drink a glass of (lukewarm) water. Enjoy your meals in the compans of friendly people.
- ✓ Eat only as much as will leave your comfortably full.
- ✓ Allow your digestive system to rest for at least 4 hours between meals.
- ✓ Have dinner no later than 3 hours before bedtime.

Relaxation habits

- ✓ Take 5 minutes each morning to consciously start the day.
- ✓ Take enough time for relaxation and rest and incorporate breaks into your daily routine.
- ✓ Several times a day pause for a minute. Close your eyes. Take several deep breaths and let go.
- ✓ Go to bed and get up at the same times each day.

Physical activity habits

- ✓ Avoid sitting for long periods of time and exercise for at least 30 minutes every day - preferably outdoors.
- ✓ Choose a kind of physical activity that you enjoy and proceed at your own pace.

Socializing habits

- ✓ Take some time off several times a week for activities with friends and family.
- ✓ Do something good for others more often …
- ✓ … and allow yourself to receive support and enjoy nice gestures from others.
